# The enigmatic helicase DHX9 as a candidate prognostic biomarker for resected pancreatic ductal adenocarcinoma

**DOI:** 10.3389/fonc.2022.1066717

**Published:** 2022-12-12

**Authors:** Le-gao Chen, Ying Cui, Wei-qin Lu, Hao Wu, Jin-song Jiang, Ke-feng Ding

**Affiliations:** ^1^ Department of Colorectal Surgery and Oncology, Key Laboratory of Cancer Prevention and Intervention, Ministry of Education, The Second Affiliated Hospital, Zhejiang University School of Medicine, Hangzhou, Zhejiang, China; ^2^ Cancer Center, Zhejiang University, Hangzhou, Zhejiang, China; ^3^ General Surgery, Cancer Center, Department of Vascular Surgery, Zhejiang Provincial People’s Hospital (Affiliated People’s Hospital, Hangzhou Medical College), Hangzhou, Zhejiang, China; ^4^ Cancer Center, Department of Nuclear Medicine, Zhejiang Provincial People’s Hospital (Affiliated People’s Hospital, Hangzhou Medical College), Hangzhou, Zhejiang, China

**Keywords:** pancreatic ductal adenocarcinoma, DEAH-box helicase 9, prognosis, recurrence, nomogram

## Abstract

**Background:**

Pancreatic ductal adenocarcinoma (PDAC) remains one of the most lethal malignancies, and current therapies have limited efficacy on PDAC. The DEAH-box helicase 9 (DHX9) is widely reported to influence cell biological behavior *via* regulating DNA replication, genomic stability, transcription, translation, and microRNA biogenesis. However, the prognostic role of DHX9 in PDAC remains unclear. Thus, the objective of this study is to investigate the prognostic value of DHX9 expression in PDAC patients.

**Methods:**

Tumor specimens from PDAC patients with surgical resection were obtained, and DHX9 was stained and analyzed in this study. Univariate and multivariate Cox regression analyses were utilized to identify independent risk factors of overall survival (OS) and recurrence-free survival (RFS). The prognostic nomograms for predicting OS and RFS were established to obtain superior predictive power.

**Results:**

Among the enrolled 110 patients, 61 patients were identified as having high expression of DHX9. The correlation analysis revealed that higher DHX9 expression in PDAC was prone to have advanced N stage (*p* = 0.010) and TNM stage (*p* = 0.017). For survival, the median OS (21.0 vs. 42.0 months, *p* < 0.001) and RFS (12.0 vs. 24.0 months, *p* < 0.001) of patients in the high DHX9 group were significantly shorter than those in the low DHX9 group. Within the univariate and multivariate analyses, American Joint Committee on Cancer (AJCC) N stage (*p* = 0.036) and DHX9 expression (*p* = 0.041) were confirmed as independent prognostic factors of OS, while nerve invasion (*p* = 0.031) and DHX9 expression (*p* = 0.005) were independent prognostic factors of RFS. Finally, the novel prognostic nomograms for OS and RFS were established and showed superior predictive accuracy.

**Conclusion:**

This study identified the independent prognostic value of DHX9 for RFS and OS in resected PDAC patients, and higher DHX9 expression was prone to have an earlier recurrence and shorter OS. Therefore, DHX9 may be a promising and valuable biomarker and a potential target for treating PDAC. More accurate and promising predictive models would be achieved when DHX9 is incorporated into nomograms.

## Introduction

Pancreatic ductal adenocarcinoma (PDAC) is a deadly malignancy in the digestive system, with a 5-year overall survival (OS) limited to 11% (1). Although great advancements in treatment strategies for cancers including chemotherapy, radiotherapy, and immunotherapy have been realized, the improvements in PDAC treatment remain at a low speed (2). Surgical resection remains the only potential treatment to cure PDAC now, but the survival of PDAC patients receiving radical resection is still unsatisfactory (3). Thus, it is necessary to investigate novel biomarkers and potential targets for treatment that are highly efficient and technically feasible to predict the prognosis of radically resected PDAC patients and develop novel therapeutic strategies.

Nucleic acid-sensing pattern recognition receptors (PRRs), widely considered as a series of innate immune receptors that recognize DNA or RNA, have been reported to participate in the progression of many cancers (4, 5). DExD/H box nucleic acid helicases including DEAD-box helicase 60 (DDX60), DEAH-box helicase 36 (DHX36), DEAD-box helicase 21 (DDX21), and DEAH-box helicase 9 (DHX9) are one group of the nucleic acid-sensing PRRs, as well, which are emerging as important regulators of many cellular processes in cancers (6). The DHX9, well-known as nuclear DNA helicase II or RNA helicase A (RHA), has been elucidated to participate in regulating genomic stability, transcription, translation, DNA replication, RNA processing and transport, and microRNA biogenesis (7).

In cancers, DHX9 has been widely reported to promote proliferation, invasion, and metastasis of cancer cells and significantly correlated with poor prognosis, including prostate cancer (7), non-small-cell lung cancer (NSCLC) (8), and hepatocellular carcinoma (HCC) (9), which may function as epigenetic regulation of gene expression program (7, 10) and influence in mRNA stability (11). In addition, DHX9 was also reported to interact with many transcription factors and influence signaling transformation (12). There were few studies about the role of DHX9 in PDAC. Only a bioinformatics analysis by Zhou et al. (13) reported that genomic loci of four downregulated circRNAs including hsa_circ_0049392, hsa_circ_000691, hsa_circ_0001626, and hsa_circ_0005203 may be controlled by DHX9 directly, which implied as potential biomarkers for PDAC. However, the specific prognostic role of DHX9 in PDAC patients after radical resection remains unclear.

In our study, we aim to clarify the prognostic role of DHX9 in PDAC. Our results revealed that the higher DHX9 expression in PDAC was remarkably prone to have higher TNM stage and lymph node metastasis. Moreover, PDAC patients with higher DHX9 expression were significantly correlated with poorer survival and earlier recurrence, and DHX9 expression in PDAC was verified as an independent risk indicator for tumor recurrence and survival. Our findings implicated that DHX9 would be a promising target for PDAC treatment.

## Methods

### Patient selection

A total of 110 tumor tissues pathologically confirmed as PDAC from patients undergoing radical surgery at our hospital from January 2013 to December 2017 were involved in this study. All the clinicopathologic data of these patients including age, gender, primary site, differentiation, T stage, N stage, TNM stage, nerve invasion, microvascular invasion, carbohydrate antigen 19-9 (CA19-9), carcinoembryonic antigen (CEA), and albumin were collected, and all patients were followed up until 30 June 2022.

### Tissue microarray and immunohistochemistry stain

Formalin-fixed, paraffin-embedded (FFPE) blocks from 110 PDAC patients after surgery were completed, and then after careful selection and review, representative tumor areas in one core (diameter, 3.0 mm) per PDAC patient were punched out to make the tissue microarray (TMA).

The TMA sections were dewaxed with xylene, dehydrated with ethanol, and then soaked with 3% hydrogen peroxide for 10 min. Next, sodium citrate buffer (pH 6.0) was used for antigen retrieval *via* heating for 20 min. When they were naturally cooled to room temperature, they were dipped in distilled water for 10 min thrice. Another 30 min was used to block the sections with 10% fetal bovine serum, and then the sections were incubated with primary anti-DHX9 antibody (Abcam, Cambridge, UK) overnight. After that, the horseradish peroxidase (HRP)-conjugated second antibody was incubated. Finally, diaminobenzidine was used for coloration and hematoxylin for counterstaining.

After dehydration and sealing, the sections were observed with an optical microscope. According to the following criteria, the expression of DHX9 was scored within the TMAs. 1) The percentage of positive staining was scored as 0, 1, 2, 3, and 4, corresponding to no positive stained cells, 1%–25% positively stained cells, 26%–50% positively stained cells, 51%–75% positively stained cells, and 76%–100% positively stained cells, respectively (2). The intensity of staining was scored as 0, 1, 2, and 3, corresponding to negative, weakly positive, moderately positive, and strongly positivity, respectively. The histochemistry score (H-score, 0–12) was calculated by multiplying the staining intensity score with the percentage of positive staining score. Accordingly, H-score < 6 was defined as low expression, while H-score ≥ 6 as high expression. All these were assessed by two experienced pathologists.

### The human protein atlas and the cancer genome atlas databases

The protein expression of DHX9 in PDAC within the Human Protein Atlas (HPA) website was searched in the website https://www.proteinatlas.org/ENSG00000135829-DHX9/pathology/pancreatic+cancer, survival analysis was performed according to the optimal grouping based on the DHX9 expression, and typical images of DHX9 expression were downloaded from the website.

### Follow-up

All patients received postoperative adjuvant chemotherapy and regular follow-ups. Enhanced abdominal computed tomography (CT) or magnetic resonance imaging (MRI) scans were performed every 6 months routinely, and physical and laboratory examinations were evaluated every 3 months for the first 2 years, every 6 months for the next 3 years, and once a year afterward. The OS was calculated as the period between surgery and death or last follow-up, and recurrence-free survival (RFS) was calculated as the period between surgery and recurrence, death, or last follow-up. This study was approved by the Ethics Committee of the Zhejiang Provincial People’s Hospital. All patients provided signed informed consent before the operation.

### Statistical analysis

Statistical analysis was performed using the R and SPSS programs. The associations between DHX9 and clinicopathologic features were analyzed using Pearson’s chi-squared test or Fisher’s exact test as appropriate. The Kaplan–Meier analyses were applied to the survival curves with the log-rank test. Univariate and multivariate analyses were performed with Cox proportional hazards regression model for determining the independent prognostic factors. The novel prognostic nomograms were operated by the rms package and evaluated by concordance index (C-index) and calibration curves. A *p*-value of less than 0.05 was regarded as statistically significant.

## Results

### Patients’ characteristics

Among the enrolled cohort, the median age was 64.0 (interquartile range (IQR), 56.8–70) years, and 66.4% (73 out of 110) of patients were male. As shown in **Table 1**, the tumor lesion was located in the pancreatic head of 61 patients (55.5%) who received pancreaticoduodenectomy, while the others received distal pancreatectomy with a tumor located on the pancreatic body or tail. The majority of patients had poorer tumor differentiation, and most of the patients receiving radical resection had a lower T and N stage. According to the American Joint Committee on Cancer (AJCC) 8th TNM staging system, there were 45, 45, and 20 patients belonging to stages I, II, and III, respectively. Nerve invasion within tumors was detected in most PDAC patients (94 out of 110), but the low population of patients had microvascular invasion within tumors (14 out of 110). In the preoperative setting, the serum CA19-9 level was elevated in 74.5% of patients, and the CEA level in 23.6%. Preoperative malnutrition with low albumin has occurred in 19.1% of PDAC patients.

**Table 1:** The relationships between DHX9 expression and clinicopathologic features.

Variables	Low DHX9	High DHX9	*p*-Value
	(n = 49)	(n = 61)	
**Sex**			0.538
Male/female	31/18	42/19	
**Age (years)**			0.983
<65/≥65	25/24	31/30	
**Primary site**			0.749
Head/body or tail	28/21	33/28	
**Differentiation**			0.705
I/II/III	2/17/30	2/17/42	
**T stage**			0.075
T1+T2/T3+T4	35/14	45/16	
**N stage**			**0.010**
N0/N1–2	36/13	30/31	
**TNM stage** I/II/III	26/19/4	19/26/16	**0.017**
**Nerve invasion**			0.118
No/yes	10/39	6/55	
**Microvascular invasion**			0.892
No/Yes	43/6	53/8	
**CA19-9**			0.452
<37/≥37 U/L	14/35	14/47	
**CEA**			0.069
<5/≥5 ng/ml	38/11	46/15	
**Albumin**			0.645
<35/≥35 g/L	11/38	10/51	

CA19-9, carbohydrate antigen 19-9; CEA, carcinoembryonic antigen. The bold values mean statistical significance.

### Expression of DHX9 proteins in pancreatic ductal adenocarcinoma and its associations with clinicopathologic features

**Figure 1:**
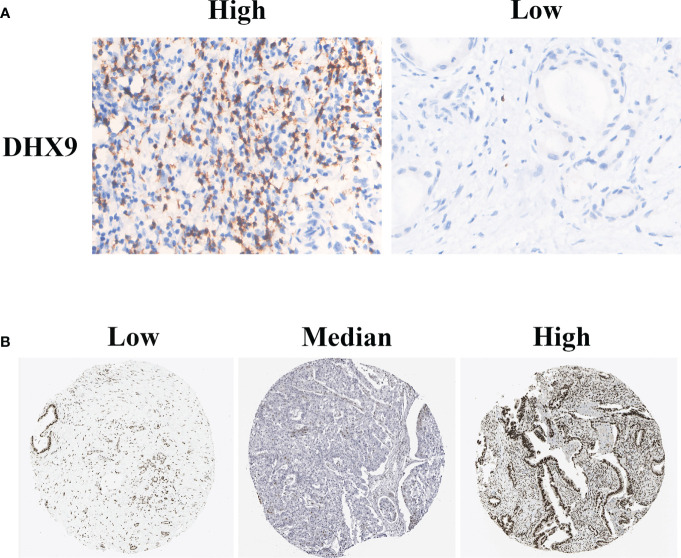
The typical immunohistochemistry staining of DHX9 in PDAC tissues from our institutional TMAs **(A)** and HPA website **(B)**. PDAC, pancreatic ductal adenocarcinoma; TMAs, tissue microarrays; HPA, Human Protein Atlas.

A representative positive and negative DHX9 staining of PDAC is presented in **Figure 1A**. The DHX9 was mainly detected on the nuclear of cancer cells and partly in the cytoplasm. According to the H-score of DHX9 expression, 61 out of 110 patients were divided into the high expression group and the other into the low expression group (n = 49). As shown in **Figure 1B**, the typical images of the low, median, and strong DHX9 expression from The Cancer Genome Atlas (TCGA) database specimens were also largely concentrated on the nuclear expression.

As shown in **Table 1**, significant associations were found between the DHX9 expression and AJCC N staging (*p* = 0.010) and TNM stage (*p* = 0.017). The higher DHX9 expression in PDAC was prone to have advanced N stage and TNM stage. However, no obvious relationship was found between DHX9 expression and the other indicators.

### Survival analysis of DHX9 in patients with pancreatic ductal adenocarcinoma

**Figure 2:**
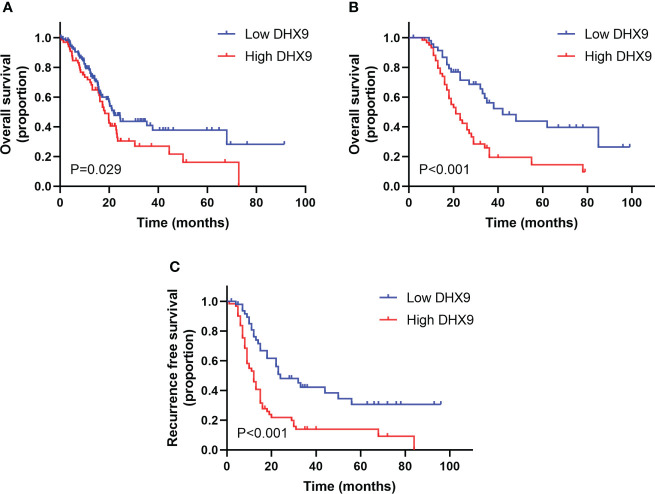
The survival analysis within the distinctive expression of DHX9 in TCGA cohort **(A)** and our institutional cohort **(B, C)**. TCGA, The Cancer Genome Atlas.

When data from TCGA database were analyzed on the HPA website, we found that the median OS in the group with low DHX9 expression was 21.7 months, significantly higher than in the group with high DHX9 expression (18.2 months, *p* = 0.029, **Figure 2A**), and the 5-year OS survival rate of the low DHX9 group was remarkably higher than that of the high DHX9 group (39% vs. 15%).

In our enrolled cohort, the total median OS and RFS were 28.0 and 15.0 months, respectively. The total 1-, 3-, and 5-year OS rates were 88.6%, 36.1%, and 28%, respectively, and the total 1-, 3-, and 5-year RFS rates were 59.3%, 26%, and 20.4%, respectively. Grouped by the DHX9 expression in PDAC, the median OS of patients was 21.0 months in the high DHX9 group, which was significantly shorter than that in the low DHX9 group (42.0 months, *p* < 0.001, **Figure 2B**). The 1-, 3-, and 5-year OS rates were 84.6%, 19.4%, and 14.5% in the high DHX9 group, respectively, which were lower than those in the low DHX9 group of 93.6%, 56.2%, and 44.0%, respectively. Similarly, the median RFS of patients in the high DHX9 group was significantly shorter (12.0 vs. 24.0 months, *p* < 0.001, **Figure 2C**). The 1- and 3-year RFS rates were 53.1% and 13.9% in the high DHX9 group, respectively, while 76.2% and 42.4% in the low DHX9 group, respectively.

Within the univariate analysis of OS, poorer tumor differentiation (*p* = 0.015), advanced AJCC T stage (*p* = 0.031), higher AJCC TNM stage (*p* < 0.001), aggressive lymph node metastasis (*p* < 0.001), aggressive nerve invasion (*p* = 0.015), and higher DHX9 expression (*p* = 0.001) were considered as significant risk factors. In the further multivariate analysis, only AJCC N stage [*p* = 0.036, hazard ratio (HR) = 2.610; 95% confidential interval (CI): 1.065–6.398] and DHX9 expression (*p* = 0.041, HR = 1.818; 95% CI: 1.024–3.225) remained as independent prognostic indicators for OS (**Table 2**). In the analysis of RFS, poorer tumor differentiation (*p* = 0.013), higher AJCC TNM stage (*p* < 0.001), aggressive lymph node metastasis (*p* < 0.001), aggressive nerve invasion (*p* = 0.004), and higher DHX9 expression (*p* = 0.001) were identified as apparent risk factors by the univariate analysis, and nerve invasion (*p* = 0.031, HR = 2.656; 95% CI: 1.095–6.442) and DHX9 expression (*p* = 0.005, HR = 2.031; 95% CI: 1.238–3.333) were finally confirmed as independent risk factors for recurrence (**Table 3**).

**Table 2:** The univariate and multivariate analyses of prognostic factors for overall survival.

Variables	Overall survival		
	Univariate *p*-Value	Multivariate *p*-Value	Multivariate HR (95% CI)
**Sex**			
Male/female	0.808	NA	
**Age (years)**			
<65/≥65	0.508	NA	
**Primary site**			
Head/body or tail	0.829	NA	
**Differentiation**			
I/II/III	**0.015**	0.293	1.331 (0.781–2.270)
**T stage**			
T1+T2/T3+T4	**0.031**	0.198	1.804 (0.735–4.426)
**N stage**			
N0/N1–2	**<0.001**	**0.036**	2.610 (1.065–6.398)
**TNM stage**			
I/II/III	**<0.001**	0.747	0.879 (0.401–1.927)
**Nerve invasion**			
No/yes	**0.015**	0.090	2.285 (0.880–5.932)
**Microvascular invasion**			
No/yes	0.295	NA	
**CA19-9**			
<37/≥37 U/L	0.275	NA	
**CEA**			
<5/≥5 ng/ml	0.310	NA	
**Albumin**			
<35/≥35 g/L	0.502	NA	
**DHX9**			
Low/high	**0.001**	**0.041**	1.818 (1.024–3.225)

CA19-9, carbohydrate antigen 19-9; CEA, carcinoembryonic antigen; NA, not available. The bold values mean statistical significance.

**Table 3:** The univariate and multivariate analyses of prognostic factors for recurrence-free survival.

Variables	Recurrence-free survival		
	Univariate *p*-Value	Multivariate *p*-Value	Multivariate HR (95% CI)
**Sex**			
Male/female	0.950	NA	
**Age (years)**			
<65/≥65	0.944	NA	
**Primary site**			
Head/body or tail	0.298	NA	
**Differentiation**			
I/II/III	**0.013**	0.206	1.355 (0.846–2.172)
**T stage**			
T1+T2/T3+T4	0.224	NA	
**N stage**			
N0/N1–2	**<0.001**	0.541	1.211 (0.655–2.239)
**TNM stage**			
I/II/III	**<0.001**	0.085	1.427 (0.953–2.137)
**Nerve invasion**			
No/yes	**0.004**	**0.031**	2.656 (1.095–6.442)
**Microvascular invasion**			
No/yes	0.778	NA	
**CA19-9**			
<37/≥37 U/L	0.410	NA	
**CEA**			
<5/≥5 ng/ml	0.828	NA	
**Albumin**			
<35/≥35 g/L	0.759	NA	
**DHX9**			
Low/high	**<0.001**	**0.005**	2.031 (1.238–3.333)

CA19-9, carbohydrate antigen 19-9; CEA, carcinoembryonic antigen; NA, not available. The bold values mean statistical significance.

### Novel prognostic nomograms for overall survival and recurrence-free survival

**Figure 3:**
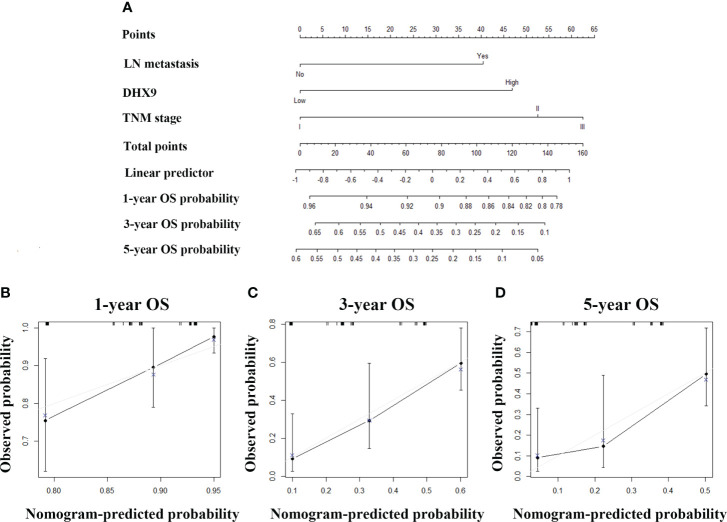
The established nomogram for overall survival **(A)** and relevant calibration curves **(B–D)**.

For purpose of accurate prediction in survival, we attempted to integrate the AJCC N staging, DHX9 expression, and TNM stage to generate a new prognostic nomogram for OS (**Figure 3A**). The C-index of the established nomogram was 0.696 (95% CI: 0.683–0.709) for OS prediction, which was higher than that of the TNM stage alone (0.654, 95% CI: 0.641–0.667). The optimal consistency was observed between the actual observation and nomogram-based prediction in 1-, 3-, and 5-year OS after surgery by the calibration curves (**Figures 3B–D**).

**Figure 4:**
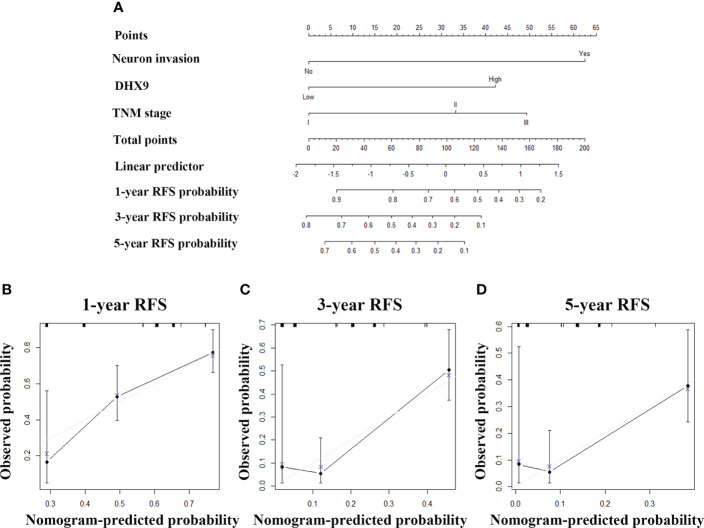
The established nomogram for recurrence-free survival **(A)** and relevant calibration curves **(B–D)**.

Then, we also tried to incorporate neuron invasion, DHX9 expression, and TNM stage into a novel prognostic nomogram for RFS (**Figure 4A**). The C-index of the established nomogram was 0.695 (95% CI: 0.683–0.707) for RFS prediction, while the C-index of the TNM stage alone was 0.622 (95% CI: 0.610–0.634), which affirmed predictive accuracy of the nomogram for RFS. The calibration plot in such a group also fitted very well between the actually observed and nomogram-predicted RFS (**Figures 4B–D**).

## Discussion

In this study, one clinical cohort of PDAC patients receiving radical surgery was analyzed. The TMA was stained with DHX9, and the DHX9 was mainly detected on the nuclear cancer cells and partly in the cytoplasm. The correlation analysis revealed that DHX9 expression in PDAC was significantly correlated with AJCC N staging and TNM stage. Next, DHX9 expression was identified as an independent risk factor for OS and RFS in resected PDAC patients with univariate and multivariate analyses, and multiple cohorts indicated that higher DHX9 expression in PDAC was prone to have an earlier recurrence and shorter OS.

DHX9, as an NTP-dependent helicase protein, plays a vital role in varieties of cellular processes, and aberrant DHX9 expression participates in multiple human diseases (14, 15) including various cancers, e.g. colorectal cancer (CRC) (16), hepatocellular carcinoma (9), and prostate cancer (7). In a variety of clinical and translational studies, DHX9 was confirmed as a prognostic gene for cancers. Recently, Lai et al. (17) reported that a total of 12 HCC-associated pyroptosis-related genes (HPRGs) including DHX9 were significantly related to poor survival, and a further five-gene risk model including DHX9 was established for prognostic prediction. As the same HCC, the DHX9-related gene prognostic model was established in gastric cancer and showed superior risk stratification (18). In patients with prostate cancer, the high DHX9 expression was significantly associated with shorter disease-free survival (DFS), which may indicate the clinical relevance of DHX9 function in prostate carcinogenesis (7). Meanwhile, DHX9 was found overexpressed in cell lines and clinical CRC tissues, and the upregulation of DHX9 in CRC patients was obviously associated with poor prognosis (16). In our study, the DHX9 expression in PDAC was found to be significantly correlated with AJCC N staging and TNM stage, which indicated that DHX9 may represent an aggressive phenotype. The same results were obtained in HCC that the high DHX9 expression was obviously correlated with metastasis, vascular invasion, and TNM stage and was verified as an independent adverse prognostic indicator (19). Additionally, Liu et al. (20) found that DHX9 in mature B cell may be a dynamic network biomarker before lymph node metastasis in CRC, which may be consistent with our findings that DHX9 expression was remarkably associated with lymph node metastasis.

In mechanism, the DHX9 can be regulated by different stimulators and then regulate various signaling. In triple-negative breast cancer (TNBC), Wang et al. (21) found that DHX9 may result in the decrease of circRNA-CREIT by interacting with the flanking inverted repeat Alu (IRAlu) sequences and inhibiting back-splicing, and then the aberrantly downregulated circRNA-CREIT was significantly correlated with poor prognosis, which may function as promoting HACE1-mediated PKR degradation and then suppressing the PKR/eIF2α signal and stress granules formation. In esophageal squamous carcinoma cells (ESCCs), Shan et al. reported that small nucleolar RNA 42 (SNORA42) could inhibit DHX9 from being ubiquitinated and degraded by reducing DHX9 transports into the cytoplasm and then increased DHX9 expression activated NF-κB signaling *via* DHX9/p65 axis to promote ESCC proliferation and migration. In the NSCLC, studies reported that the DHX9-related circular RNAs (circRNAs) such as circ-PTEN (22) and circDCUN1D4 (23) were significantly decreased in the NSCLC tissues that promoted its progression and metastasis, which functioned by circ-PTEN/miR-155/miR-330-3p/PTEN and circDCUN1D4/HuR/TXNIP regulatory axes, respectively. The DHX9 could also directly interact with long non-coding RNAs (lncRNAs) including SH3PXD2A-AS1 to enhance FOXM1 expression and finally promoted NSCLC cell proliferation and progression (8). In addition, the epigenetic “reader” Tudor domain-containing protein 3 (TDRD3) could directly interact with DHX9 by its Tudor domain, which was critical for DHX9 recruitment to target gene promoters, where it resolved R-loops with a helicase activity-dependent way to promote various gene expression (24).

In addition to the role of DNA replication, genomic stability, and transcription, the DHX9 may also participate in the shaping of the tumor microenvironment. Liang et al. (18) reported significant infiltrations of macrophages, dendritic cells, and neutrophils in gastric cancer patients with high DHX9 expression. In HCC, the six DEAH-box RNA helicases in the contrasted risk model were substantially associated with immune inhibitors and innate immune cell infiltrations (9). In addition, Jiao et al. (25) also found that T cell-specific deletion of DHX9 led to impaired CD8^+^ T-cell survival, viral clearance, and effector differentiation in COVID-19 patients.

Despite these findings, some limitations remained in our study. First, we only used a single cohort with retrospective data to present the prognostic role of DHX9 in PDAC, so further verifications are required. Second, the potential mechanism of DHX9 was not investigated in our current study, so further experimental work will be performed to illustrate the specific role of DHX9 in PDAC. Third, an external, multicenter, large cohort and prospective clinical trial remains required for further validation of the established nomograms.

## Conclusion

Our results revealed that the DHX9 expression in PDAC was significantly associated with lymph node metastasis and tumor TNM stage. The DHX9 expression was identified as an independent risk factor of RFS and OS in resected PDAC patients, and higher DHX9 expression was prone to have an earlier recurrence and shorter OS. Therefore, DHX9 may be a promising and valuable prognostic biomarker and a potential target for PDAC treatment. More accurate and promising predictive models would be achieved when DHX9 is incorporated into nomograms.

## Data availability statement

The data presented in the study are deposited in the Human Protein Atlas repository (https://www.proteinatlas.org/ENSG00000135829-DHX9/pathology/pancreatic+cancer) with no access restriction, and the raw data supporting the conclusions of this article will be made available by the authors upon reasonable request.

## Ethics statement

The studies involving human participants were reviewed and approved by Zhejiang Provincial People’s Hospital. The patients/participants provided their written informed consent to participate in this study.

## Author contributions

Study concept and design: K-FD, J-SJ, and L-GC. Acquisition of data: L-GC, YC, W-QL, and HW. Statistical analysis: L-GC. Drafting of the manuscript: L-GC and YC. Critical revision of the manuscript for important intellectual content: L-GC, YC, W-QL, HW, J-SJ, and K-FD. All authors contributed to the article and approved the submitted version.
